# Phytoplankton and benthic infauna responses to aeration, an experimental ecological remediation, in a polluted subtropical estuary with organic-rich sediments

**DOI:** 10.1371/journal.pone.0280880

**Published:** 2023-01-24

**Authors:** Xiao Ma, Austin Fox, Stacey Fox, Kevin B. Johnson

**Affiliations:** 1 Key Laboratory of Tropical Marine Bio-Resources and Ecology, South China Sea Institute of Oceanology, Chinese Academy of Sciences, Guangzhou, China; 2 Department of Ocean Engineering and Marine Sciences, Florida Institute of Technology, Melbourne, Florida, United States of America; Universidad Federal de Minas Gerais, BRAZIL

## Abstract

Fine-grained organic-rich sediments (FGORS) are accumulating in estuaries worldwide, with multi-faceted negative ecosystem impacts. A pilot experiment was carried out in a residential canal of the Indian River Lagoon estuary (IRL, Florida, USA) using an aeration treatment intended to mitigate the harmful ecological effects of organic-rich sediment pollution. Planktonic and benthic communities were monitored, and environmental data collected throughout the aeration process. Results were compared against control conditions to evaluate the efficacy of aeration in the mitigation of FGORS. During the aeration process, hurricane Irma impacted the study area, bringing heavy rainfall and spawning a brown tide event (*Aureoumbra lagunensis*). The overall thickness and volume of FGORS, and the organic content of surface sediments did not change during the aeration treatment. Dissolved oxygen was higher and ammonium concentrations were lower in aeration canal bottom water compared to the control canal. During treatment, aeration did facilitate benthic animal life when temperatures dropped below 25°C, likely due to water column mixing and the increased capacity of water to hold dissolved gasses. In general, aeration did not significantly change the planktonic community composition relative to the control canal, but, during the post-bloom period, aeration helped to weaken the brown tide and phytoplankton densities were 35–50% lower for *A*. *lagunensis* in aeration canal surface water compared to the control canal. Aeration has important management applications and may be useful for mitigating algal blooms in flow-restricted areas and promoting benthic communities in cooler environments.

## Introduction

Low-energy estuaries, such as the Indian River Lagoon (“IRL”, Florida) have accumulated fine-grained, organic-rich sediments (FGORS). FGORS have been locally termed “muck” [1], operationally defined as > 10% organic matter, > 60% silt and clay, and > 75% water by weight. IRL FGORS thicknesses have increased an average of 67% over three decades, and these sediments are ubiquitous in channels and canals [1–3]. FGORS sources vary, but include eroded soil, construction run-off, sewage discharge, decomposed lawn clippings, dead organisms, and municipal or industrial runoff [4]. FGORS supply > 50% of total IRL water column nitrogen (15 tons km^-2^ year^-1^) [5, 6], and these fluxes perpetuate eutrophication and harmful blooms [7]. As senescing blooms decompose, bacterial respiration depletes oxygen, stressing or suffocating seagrasses and other aquatic life [8]. Bloom material accumulates in sediments, creating a feedback loop between FGORS and harmful algal blooms.

Remediation techniques are being developed to treat eutrophic conditions in polluted estuaries around the world, with approaches including environmental dredging, enhanced circulation, and aeration [5, 9–11]. Aeration has been proposed as a less expensive tool compared to dredging for reducing organic sediments and hypoxia [12]. It mixes the water column, increases dissolved oxygen (DO) concentrations and accelerates aerobic decomposition of organic matter [13–15]. For example, the cost of aerating our study canal was $10,000–15,000 USD for initial setup plus ~3,500 USD annually for electricity (Allied Group USA, *personal communication*). In contrast, the cost of environmental dredging is $50–75 USD per cubic meter (i.e., $200,000 for ~4,000 m^3^ of muck from the same canal). Artificial aeration has been used in lakes and aquaculture ponds to enhance water quality, while little *in situ* data is available to inform predictions of ecosystem outcomes for estuaries undergoing aeration [12, 14–19]. Those freshwater studies focus on chemical reactions and microbial processes [14, 16–19]. The few studies of estuarine aeration are limited to laboratory or mesocosm investigations [20–24], with the exception of a few *in situ* studies examining water quality only [13, 25, 26]. Many of the aforementioned aeration studies showed positive, albeit site-specific, ecological effects. For example, aeration may increase dissolved oxygen [13, 14] or accelerate FGORS decomposition [15].

This study of aeration in an estuarine residential canal was conducted to provide data in a replicated and controlled *in-situ* environment. Biological and environmental responses were compared between the aerated canal and an adjacent non-aerated control canal of similar structure and circulation. In this *in situ* estuarine study, we hypothesized that 1) aeration would reduce hypoxia and eutrophic nutrient levels; 2) aeration would change phytoplankton and benthic infauna abundances, diversity and community compositions.

## Materials and methods

### Study sites and aeration treatment

**Fig 1:**
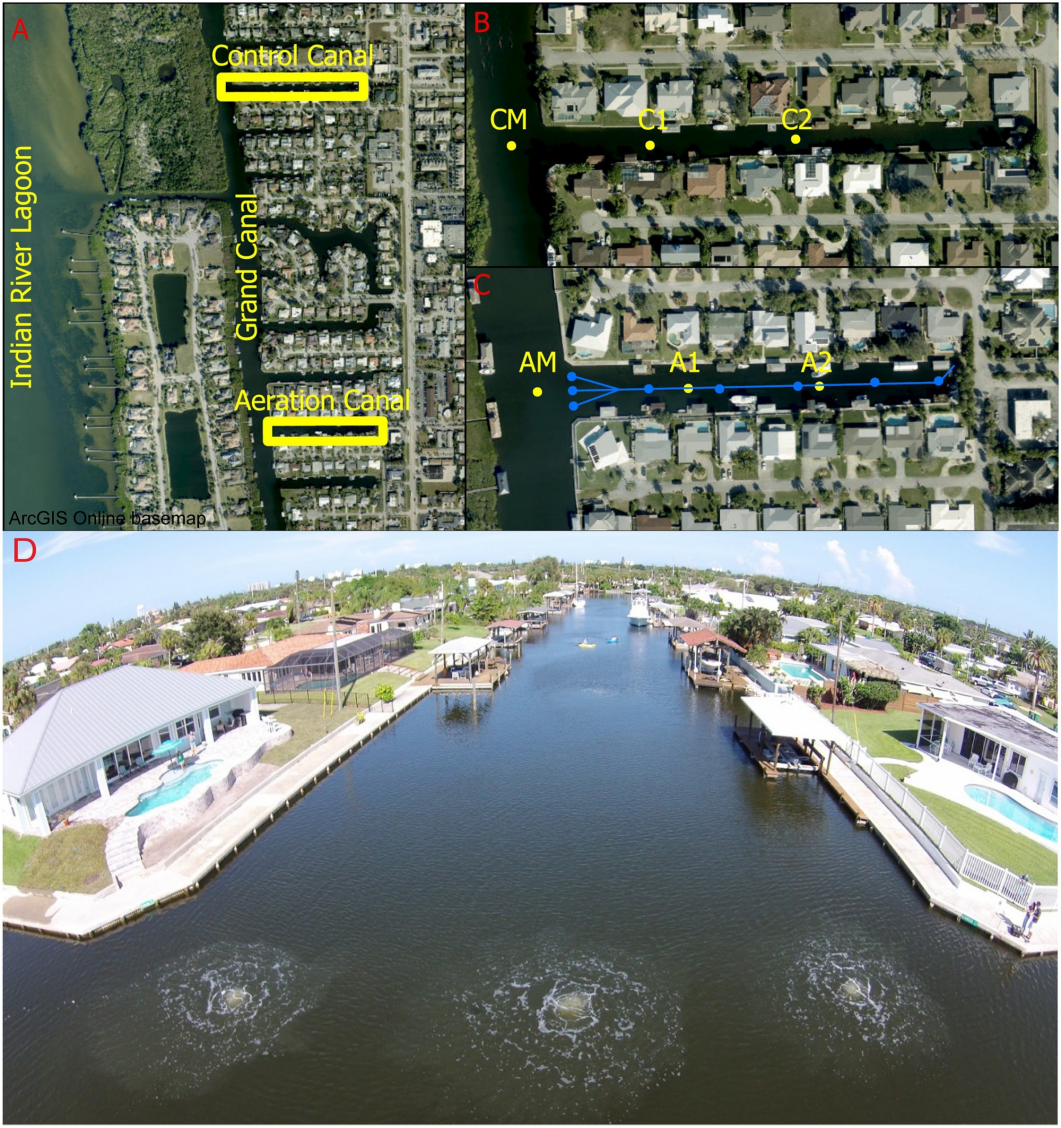
Study locations in (A) Aeration Canal and Control Canal, adjacent to the Grand Canal in Satellite Beach. (B) Control Canal sampling stations (yellow dots: C1 = Control 1; C2 = Control 2). (C) Aeration Canal sampling stations (yellow dots: A1 = Aeration 1; A2 = Aeration 2). CM (Control mouth in Grand Canal) and AM (Aeration mouth in Grand Canal) are intermedia sites in the Grand Canal; blue dots are aeration diffusers and blue lines are sunken aeration tubing. The maps for (A), (B) and (C) were generated from ArcGIS Online basemap [43] (D) A picture of aeration canal with diffusers running.

This study was carried out in the mid-section of the northern IRL system located on the east coast of central Florida, USA. No permit was required for the study since the experiment was conducted in the public waterways. It is in a humid subtropical climate zone with a rainy season from May through September, and a dry season from October to late April. The average annual temperature and precipitation are 22.2°C and 136.4 cm, respectively. The experiment was conducted in residential canals connected to the larger estuary (IRL) through the Grand Canal with no direct freshwater discharge into the experimental area (Fig 1A, 1C and 1D). Residential canals are long rectangular canals lined with seawalls. They constitute the back yards of single-family houses abutting the canal for convenient estuary access. One canal was aerated and another, 800-m away, served as control. The control canal was selected for its geomorphological and hydrological similarities to the aerated canal (Fig 1A and 1B). One sampling station near the mouth of each canal, but in the Grand Canal, was selected as an intermediate site for comparison (Fig 1B and 1C). The aeration system for this 6070.3 m^2^ canal consisted of five, 30.48 cm^2^ micro-porous diffusers placed on the benthos, amongst FGORS, with 50-m between diffusers (Fig 1C and 1D). Three additional diffusers were placed next to the fifth diffuser at the mouth of the canal to establish a fine bubble retention curtain to limit the loss or gain of FGORS (Fig 1C and 1D). Diffuser placement and pump size were selected based on standard industry practices. Two 1.5 hp air pumps yielded 2.47×10^−4^ hp per m^2^. Diffuser spacing was determined based on the depth of the canal. At 2.5 m deep, each diffuser circulates ~1011.7 m^2^, therefore requiring five evenly spaced diffusers for the 6070.3 m^2^ treatment area (Allied Group USA, *personal communication*). Diffusers were under continuous operation from August 2017 to June 2018, but shut down for a week during Hurricane Irma. Biological and water quality monitoring were carried out before (8/14/2017) and during aeration (9/22/2017, 11/21/2017, 2/21/2018, 4/24/2018 and 6/25/2018). Biological and water quality sampling locations are indicated in Fig 1, with sampling details below. Sediment surveys were carried out on 7/26/2017 (pre-aeration) and 8/14/2018 (post-aeration). Hurricane Irma passed through the area in September 2017 and another sediment survey was carried out eleven days after the hurricane to evaluate storm sediment impacts. The sediment survey and sampling locations are indicated in S1 Fig.

### Phytoplankton collection and laboratory processing

The canal water column averaged 2.5 m in depth. Phytoplankton samples were collected using a peristaltic pump with acid-washed Tygon tubing at specified depths (0.5m below the surface and 0.5m above the bottom) at the 6 stations (Fig 1B and 1C). Three replicates were taken at each sampling depth at each station. Each sample was split into two subsamples. One subsample (30 ml) was filtered (53-μm), kept on ice, and analyzed using flow cytometry (BD Accuri C6) within 12 hrs of collection. The other subsample (250 ml) was preserved in 4% formalin pending analysis and identification. Preserved samples were concentrated using the sedimentation technique described in *Standard Operating Procedure for Phytoplankton Analysis* [27]. The sedimentation process was conducted in 24-cm graduated cylinders and took 6 days. After settling, overlying water was drawn off via syringe. Concentrated samples were counted using a graduated Sedgewick Rafter counting chamber until ≥ 300 cells were counted. Phytoplankton were identified to the lowest possible taxonomic level. Flow cytometry was used to differentiate nano-phytoplankton according to their pigmentation and cell sizes [28]. Counting via compound light microscopy yielded the identities and densities of the larger micro-phytoplankton.

### Benthic infauna collection and laboratory processing

Benthic infauna sampling approach followed Poirrier et al. (2008) [29]. Three benthic infauna samples were collected randomly at each sampling location (Fig 1B and 1C) using a Wildco Petite Ponar Grab (sampling area 225 cm^2^). Sediment grab volumes, measured via graduated cylinder, enabled determination of grab penetration depth. Samples were sieved through 500-μm mesh [30] and retained organisms were frozen for lab analysis. The infauna samples were examined within one month after collection and thawed organisms retained their anatomical structure enabling confident laboratory identification. Aliquots of each sample (1/4 or 1/8) were sorted to ensure at least 100 organisms were counted in each sample. Organisms were identified to the lowest possible taxonomic level and counted via stereomicroscopy (8×-35× magnification).

### Environmental data collection methods

All environmental data were collected simultaneously with phytoplankton samples. Secchi Depths were measured using a standard black-white estuary-style Secchi disk. The disk was lowered into the water column with a graduated rope under sunlight. The Secchi Depth is the depth where underwater disk pattern differentiation is no longer possible. Temperature, salinity, pH and DO were measured using Yellow Springs Instruments ProDSS. The Sonde was factory calibrated and then calibrated following manufacturer’s guidelines immediately prior to each sampling event. Water depths and FGORS thicknesses were determined at mapped locations (~ 50 locations in each canal, S1 Fig) using a capped 4-cm diameter polyvinyl chloride (PVC) probe. The probe was first lowered through the water column to the benthic surface and water depth was recorded. The probe was then pushed into the sediment until encountering a firm bottom, and this was recorded as the total (muck plus water column) depth. The thickness of probe-penetrable sediment (muck) was calculated by subtracting the water depth from the total depth. Water depths were verified by sounding with a 20-cm disc that settled onto the surface of soft sediments. Sediment thicknesses were validated for selected sites with sediment cores. The surface areas and volumes of muck were extrapolated by contouring probe data via ArcGIS (S1 Fig). Samples for sediment quality analysis were collected during the FGORS thickness survey using a 0.15 m^2^ Ekman grab. Subsamples of the surface sediments (top 3 cm) were placed into polystyrene vials and sealed with parafilm. Samples were then weighed, frozen, freeze-dried (Labconco Freezone 6) and re-weighed to determine water content. Dried sediments were homogenized (Spex 8000 mixer mill) and organic matter content was determined via the Loss on Ignition (LOI) method (loss of mass after combustion at 450°C for 4 hours) [31].

Water samples for nutrient analyses were collected alongside phytoplankton samples using a peristaltic pump and acid-washed (HCl) Tygon tubing at multiple depths at each sampling station. Water samples were pumped into acid-washed LDPE bottles and stored on ice for transport. In the laboratory, samples were immediately vacuum-filtered through 47-mm diameter 0.4-μM pore-sized polypropylene filters. Analyses of ammonium (NH_4_+), nitrate plus nitrite (NO_3_^-^ + NO_2_^-^), total dissolved nitrogen (TDN), ortho-phosphate (PO_4_^3-^), total dissolved phosphorus (TDP) and silica were determined using a segmented continuous flow nutrient autoanalyzer (SEAL AA3) following the manufacturer’s methods. Standard reference materials were run with each batch of samples and standards were within 10% of the certified values for all analyses.

### Statistical analyses

One-way analysis of similarities (ANOSIM) based on Bray-Curtis similarities was used to detect differences in phytoplankton assemblages between and among different sites, and before and after aeration (α = 0.05, R version 4.1.3). Similarity percentage analysis (SIMPER) was used to determine which species contributed to observed differences (R version 4.1.3). Phytoplankton density data were square-root transformed for ANOSIM and SIMPER tests. Environmental parameters, benthic infauna densities, Shannon’s Diversity Indexes and *A*. *lagunensis* cell densities were compared between aeration and control canals using 2-way ANOVA and Fisher’s LSD *post-hoc* pairwise comparisons (α = 0.05, GraphPad Prism v9). Spearman’s correlation analyses between benthic infauna diversity indexes and abundances and environmental parameters were performed for all sampling stations (α = 0.05, R version 4.1.3).

## Results

### Phytoplankton community—changes during aeration

**Fig 2:**
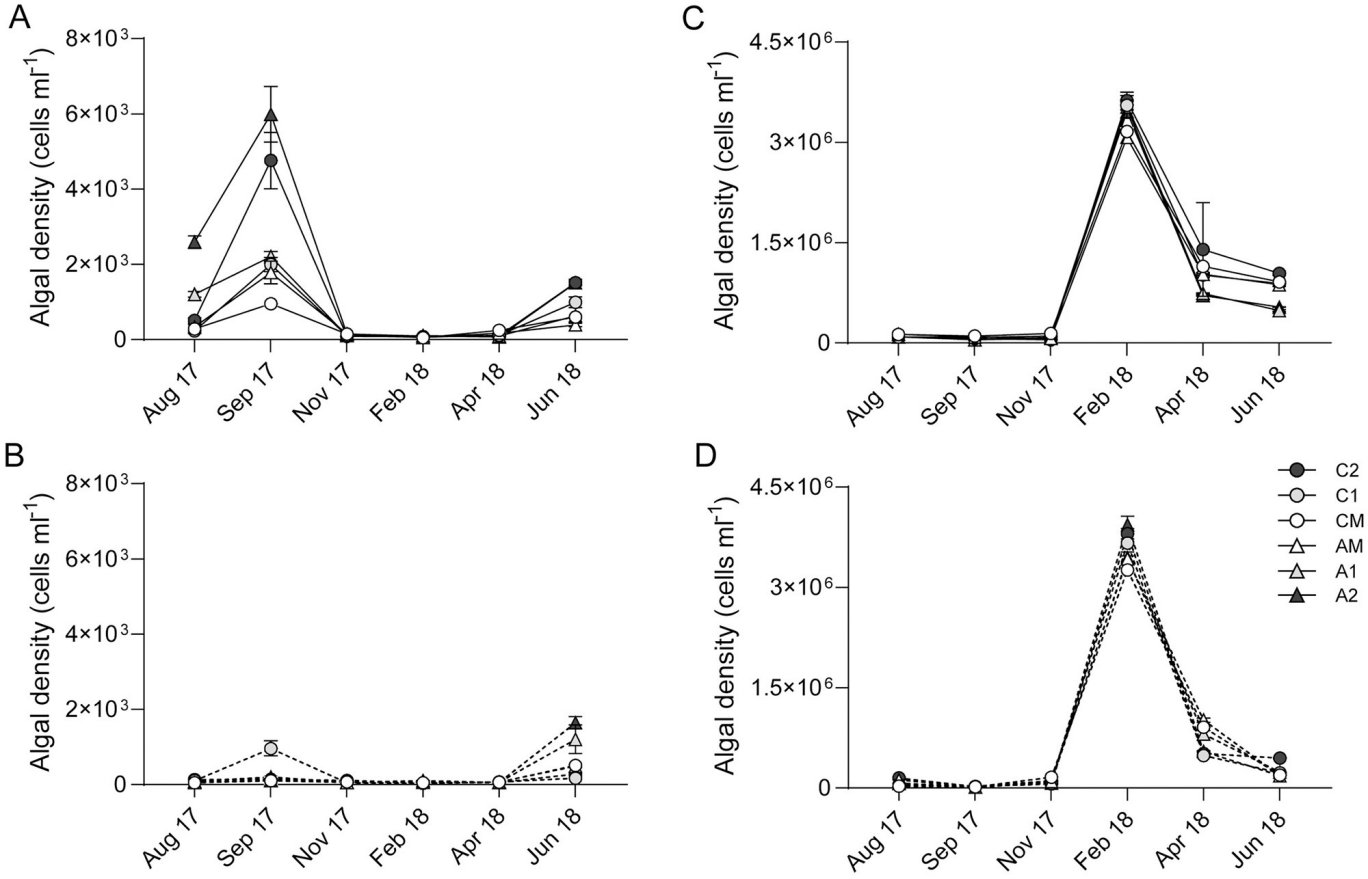
(A) Micro-phytoplankton and (B) Nano-phytoplankton densities (Mean ± SD) from surface and bottom waters in aeration canal (A1, A2), control canal (C1, C2) and Grand canal (CM, AM) in different months.

Fifty-two micro-phytoplankton taxa, mostly dinoflagellates, were found in canals (S1 Table). Twenty-one nano-phytoplankton taxa were found in canals, including 11 groups of cyanobacteria (S1 Table). Two algal blooms were observed during the study. *Scrippsiella sp*., a dinoflagellate, formed a small bloom in surface water in September 2017 with an average density > 1×10^3^ cells ml^-1^ in all canals (Fig 2A). *A*. *lagunensis*, notoriously referred to as “brown tide”, bloomed from February 2018 to June 2018, with the highest density being 3.5 × 10^6^ cells ml^-1^ (Fig 2C and 2D).

**Fig 3:**
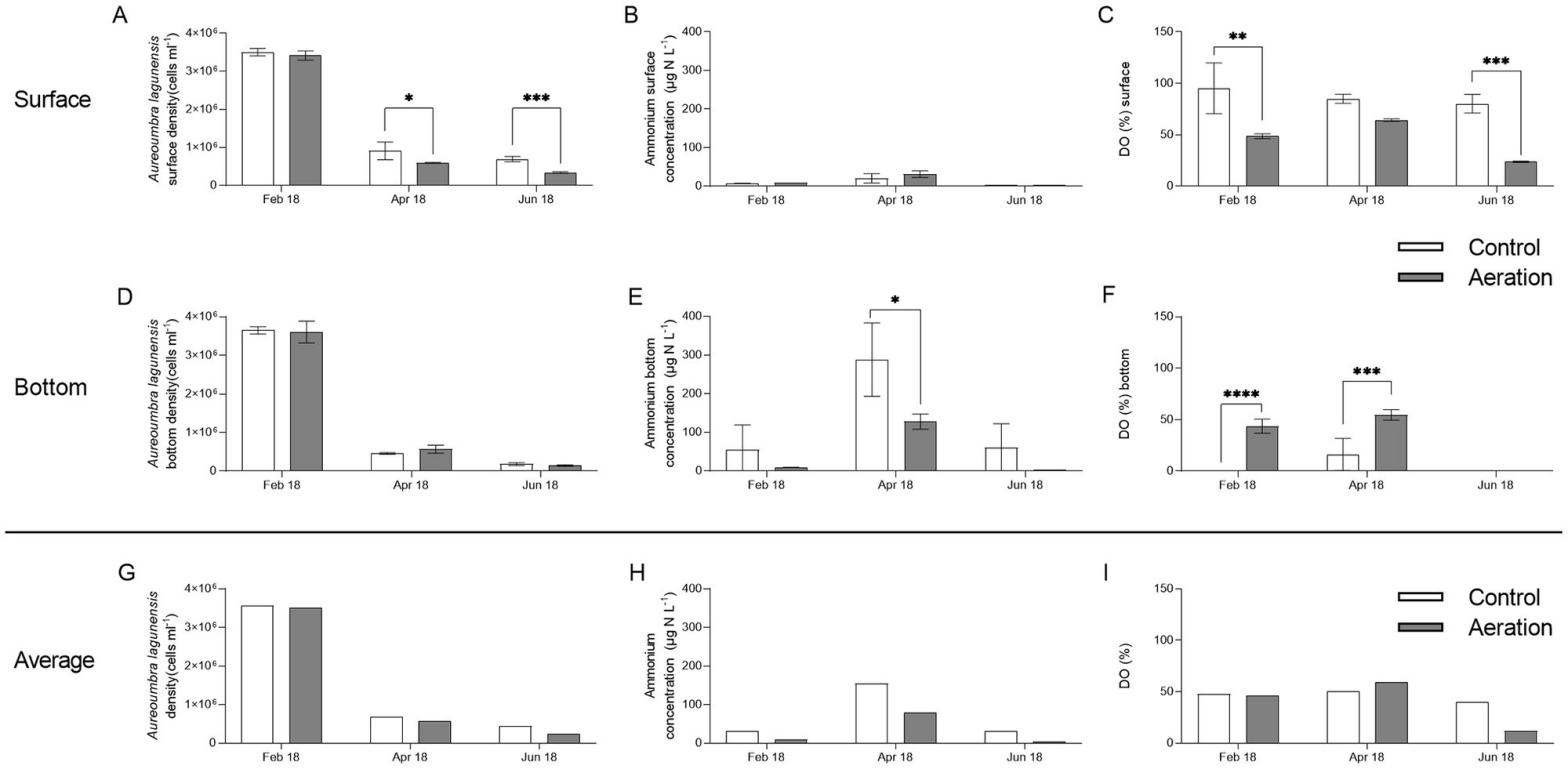
Surface water (A) *Aureoumbra lagunensis* cell densities, (B) ammonium concentrations and (C) DO (%) in control vs. aeration canals from February to June 2018. Bottom water (D) *A*. *lagunensis* cell densities, (E) ammonium concentrations and (F) DO (%) in control vs. aeration canals from February to June 2018. Average (G) *A*. *lagunensis* cell densities, (H) ammonium concentrations and (I) DO (%) in control vs. aeration canals from February to June 2018. Different significance levels were determined via 2-way ANOVA and Fisher’s LSD *post-hoc* pairwise comparisons (* *p* < 0.05, ** *p* < 0.01, ***p < 0.001, ****p < 0.0001).

Nano-phytoplankton dominated all canals year-round (Fig 2C and 2D). Densities of nano-phytoplankton were 2 orders of magnitude higher than that of micro-phytoplankton during non-bloom periods, and 4–5 orders higher during the bloom (Fig 2). The main differences between the aeration and control canals were 40% and 47% fewer total cell counts for surface nano-phytoplankton in the aeration canal in April and June 2018, respectively (Fig 2C). This is mainly attributed to the cell density differences of *A*. *lagunensis* (Fig 3A). Surface water cell counts for *A*. *lagunensis* were significantly lower in the aerated canal relative to the control in April 2018 (*p* < 0.05) and June 2018 (*p* < 0.001) (Fig 3A). Meanwhile, average ammonium (Fig 3B, 3E and 3H) and surface water DO concentrations (Fig 3C) were lower in the aeration canal compared to the control from February to June 2018.

**Fig 4:**
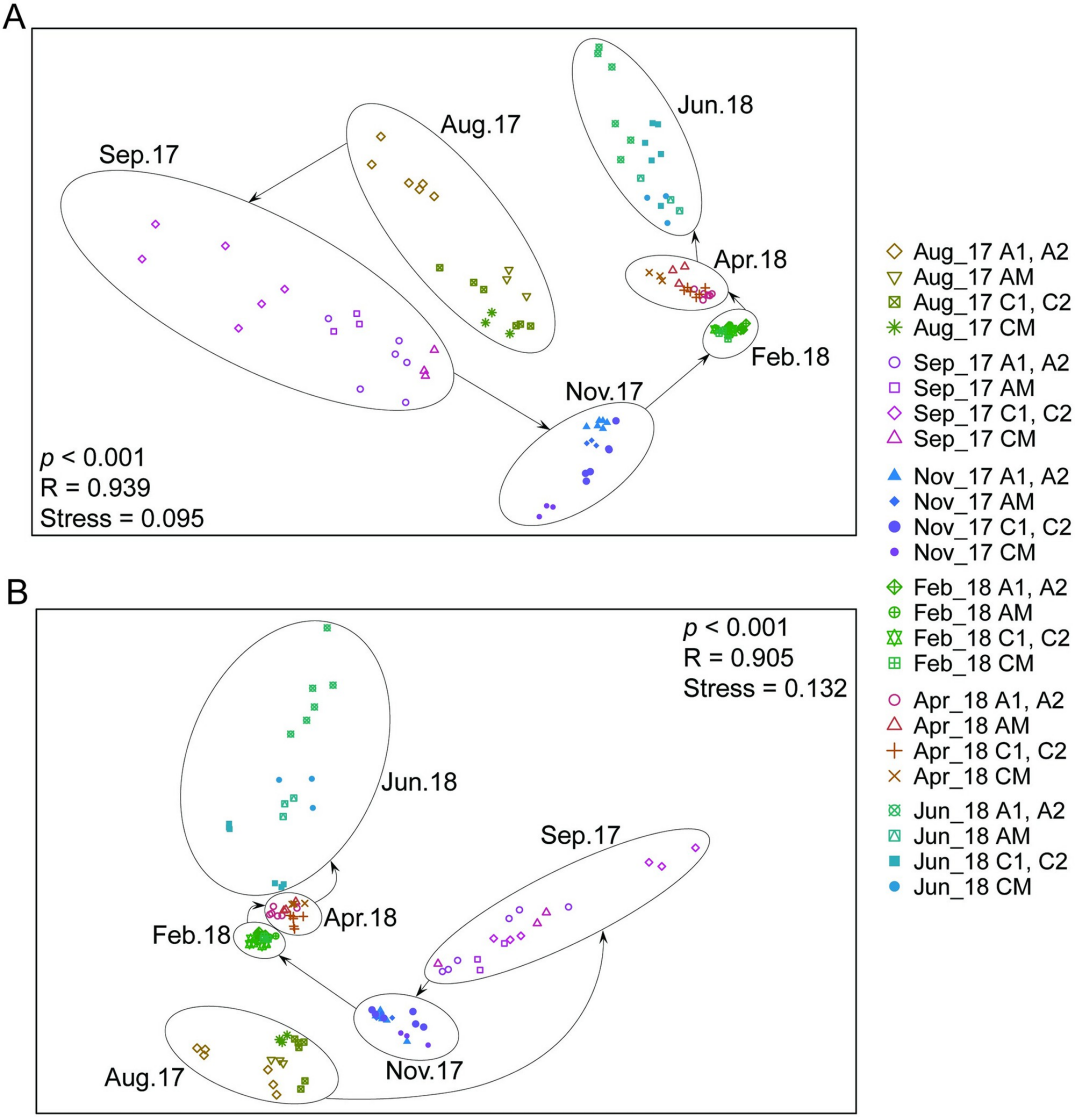
Two-dimensional nMDS ordination plots of planktonic assemblages in (A) surface and (B) bottom waters over the study period. Treatments are canal locations, with aeration canal (A1, A2), control canal (C1, C2) and Grand canal (CM, AM) in different months. The *p* and R values displayed in the figure were ANOSIM test results.

**Fig 5:**
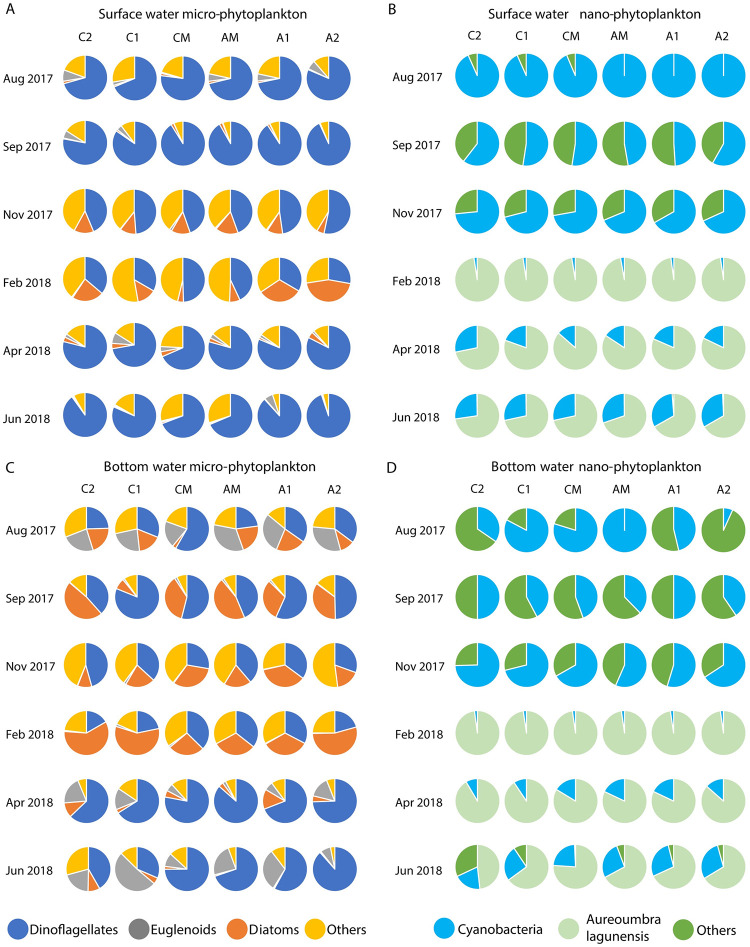
(A) Surface water micro-phytoplankton, (B) surface water nano-phytoplankton, (C) bottom water micro-phytoplankton and (D) bottom water nano-phytoplankton compositions in the control Canal (C1, C2), Grand canal (CM, AM) and aeration canal (A1, A2) in different months.

Comparing across months, temporally distinct planktonic communities developed throughout the experimental period (Fig 4). Both of the surface and bottom planktonic communities were more divergent in summer and fall, but converged in winter and spring (Fig 4). Dissimilarities among different months mainly came from nano-sized cyanobacteria and the *A*. *lagunensis* bloom (ANOSIM and SIMPER tests, *p* < 0.001, R > 0.95). After aeration (September 2017- June 2018), bottom water planktonic community assemblages from the aeration canal were more consistent with those of the Grand Canal compare to the control canal (Fig 5C and 5D). However, in general, aeration treatment differences were overshadowed by seasonal variations (Figs 4 and 5).

### Benthic infauna community—changes during aeration

**Fig 6:**
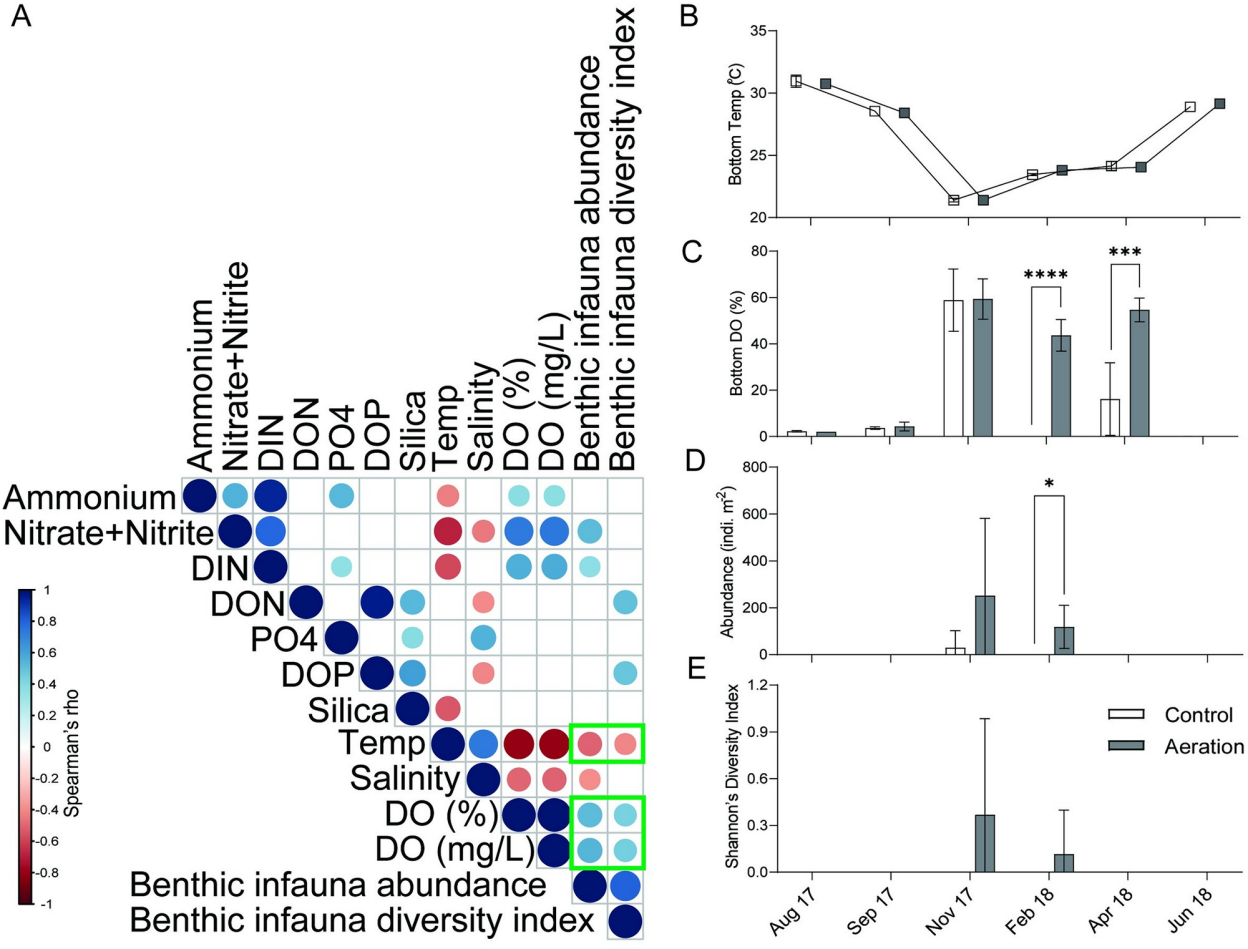
(A) Correlation analyses of benthic infauna community (both abundance and Shannon’s Diversity Index) and bottom water environmental parameters for all samples during the whole study period. The scale shows the degree of positive correlation (blue) or negative correlation (red) between two variables. Nonsignificant correlations (adjusted *p* > 0.05) are indicated by blanks. Green rectangles highlight the environmental parameters (Temperature and DO) that correlate to both benthic infauna abundance and Shannon’s Diversity Index. Comparisons of (B) bottom temperature (C) bottom DO (D) benthic infauna abundance (E) benthic infauna Shannon’s Diversity Index compared between the control and aeration canal in different months (2-way ANOVA and Fisher’s LSD *post-hoc* pairwise comparisons, where * *p* < 0.05, ***p < 0.001, **** *p* < 0.0001).

Benthic infauna were present only in late fall to early spring (Table 1). In February 2018, benthic infauna were significantly more abundant in the aeration canal relative to the control (*p* < 0.05, Fig 6D). Meanwhile, bottom water temperature was below 25°C (Fig 6B) and DO concentration was significantly higher in the aeration canal compared to the control in February 2018 (*p* < 0.0001) and April 2018 (*p* < 0.001) (Fig 6C, Table 1). Correlation analyses between benthic infauna communities and environmental parameters revealed that both abundance and diversity of benthic infauna positively correlated with DO (*p* < 0.05, rho > 0.5) and negatively correlated with temperature (*p* < 0.05, rho < -0.5) (Fig 6A).

**Table 1:** Benthic infauna species composition and abundance (individuals m^-2^) at each sampling location in different months.

	C2		C1	CM		AM		A1		A2	
Aug 2017	/		/	/		/		/		/	
Sep 2017	/		/	/		/		/		/	
Nov 2017	Unidentified Gammarid Amphipod	59	/	*Mulinia lateralis*	59	*Ctenodrilus serratus*	178	*Ctenodrilus serratus*	267	*Ctenodrilus serratus*	59
				*Ctenodrilus serratus*	59			Unidentified Gammarid Amphipod	59		
								Unidentified Clam	30		
								Nannastaciadae	30		
								Unidentified Polychaete	59		
Feb 2018	/		/	*Leptochelia dubia*	30	*Mulinia lateralis*	59	*Capitella capitata*	30	*Capitella capitata*	119
				*Mulinia lateralis*	30	Unidentified Gammarid Amphipod	89	Unidentified Polychaete	30	Unidentified Polychaete	59
						*Paradiopatra hispanica*	30				
Apr 2018	/		/	*Mulinia lateralis*	30	/		/		/	
Jun 2018	/		/	/		/		/		/	

Slash (/) indicates that there was no organism found in that month at that location.

### Environmental conditions—changes during aeration

**Fig 7:**
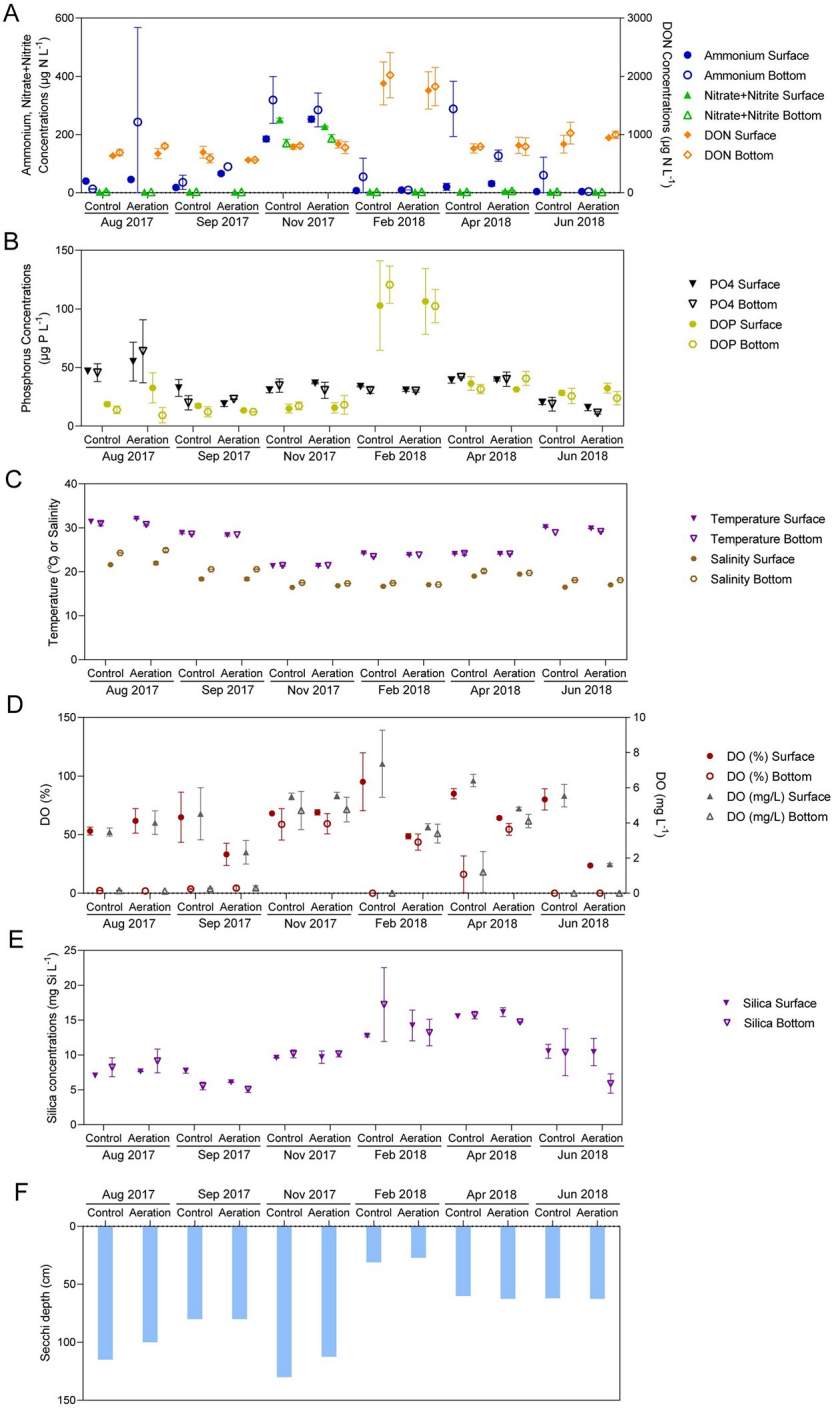
(A) nitrogen concentrations (Ammonium, Nitrate+Nitrite, Dissolved Organic Nitrogen (DON)), (B) phosphorus concentrations (Phosphate, Dissolved Organic Phosphorus (DOP)), (C) temperature and salinity, (D) Dissolved Oxygen (DO), (E) Silica concentrations and (F)Secchi depth in the control and aeration canals at both surface and bottom layers in different months.

Concentrations of ammonium and nitrate + nitrite peaked in all canals at >200 μg N L^-1^ during November 2017 following Hurricane Irma (Fig 7A). Coincident with the *A*. *lagunensis* bloom in February 2018, near-complete depletion of ammonium and nitrate + nitrite was observed in all canals. Depletion of Dissolved Inorganic Nitrogen (DIN) coincided with a sharp increase in Dissolved Organic Nitrogen (DON) (Fig 7A). Concentrations of ortho-phosphate showed peak concentrations of >40 μg P L^-1^ in all canals before aeration, and remained below 40 μg P L^-1^ during aeration (Fig 7B). Dissolved Organic Phosphorus (DOP) showed the same trend observed for DON, which peaked > 100 μg P L^-1^ in February 2018 during the *A*. *lagunensis* bloom and was around 20 μg P L^-1^ before and after the bloom period. Silica concentrations remained high during the whole sampling period (6 to 16 mg Si L^-1^) (Fig 7F).

Salinity and temperature followed similar vertical and temporal trends in all canals. Salinity stratifications were observed in summer, with maximum salinity of 25 in the bottom water column in August 2017 (Fig 7C); however, during cooler months when water temperatures were below 25°C, salinity was well-mixed. Vertically uniform salinities continued in all canals with values increasing to 19 by April 2019, after which the seasonal pattern began to repeat (Fig 7C). Temperature was uniform with depth all year in all canals with maxima of 29–32°C during August 2017 and June 2018 and minima of 21–22°C in November 2017 (Fig 7C). Prior to the onset of aeration (August 2017), bottom water was anoxic in all canals. However, by November 2017, aerated canal water had DO concentrations of ~60% saturation throughout the water column (Fig 7D). In contrast, DO was variable in the control canal, with several instances of hypoxia (Fig 7D). Bottom water DO was significantly higher in the aeration canal compared to the control in February 2018 (*p* < 0.0001) and April 2018 (*p* < 0.001) (Fig 7C, Table 2), while surface water DO was significantly higher in the aeration canal in September 2017 (*p* < 0.05), February 2018 (*p* < 0.01) and June 2018 (*p* < 0.001) (Table 2). Secchi Depths were deeper in the non-bloom periods, but only 30 cm during the *A*. *lagunensis* bloom in February 2018 (Fig 7E).

**Table 2:** Summary of outcomes of statistical comparisons of control and aeration canals as a function of environmental parameters and temporal changes.

		Aug 2017		Sep 2017		Nov 2017		Feb 2018		April 2018		Jun 2018	
		Diff.	Sig.	Diff.	Sig.	Diff.	Sig.	Diff.	Sig.	Diff.	Sig.	Diff.	Sig.
Control—Aeration	Ammonium (μg N L^-1^) Surface	-5.60		-48.06	**	-68.09	**	-1.60		-10.72		0.00	
	Ammonium (μg N L^-1^) Bottom	-229.80	*	-53.81		34.44		45.37		160.70	*	56.03	
	Nitrate+Nitrite (μg N L^-1^) Surface	0.00		0.00		23.23	****	-0.03		-2.66		0.00	
	Nitrate+Nitrite (μg N L^-1^) Bottom	1.38		0.68		-16.06	*	0.13		-3.43		1.33	
	DON (μg N L^-1^) Surface	-37.82		132.40		-47.18		120.50		-53.16		-110.70	
	DON (μg N L^-1^) Bottom	-111.30		26.52		30.84		196.80		-1.05		30.18	
	PO4 (μg P L^-1^) Surface	-8.21		13.59	*	-5.74		3.26		0.00		4.49	
	PO4 (μg P L^-1^) Bottom	-18.29		-3.09		4.08		0.44		1.70		7.43	
	DOP (μg P L^-1^) Surface	-13.94		3.98		-0.66		-3.57		4.96		-4.03	
	DOP (μg P L^-1^) Bottom	4.59		-0.01		-0.97		18.17	*	-9.14		1.86	
	Temperature (°C) Surface	-0.65	***	0.50	**	-0.05		0.35	*	0.00		0.30	
	Temperature (°C) Bottom	0.20		0.15		0.00		-0.35		0.10		-0.25	
	Salinity Surface	-0.35		0.01		-0.39		-0.36		-0.44	*	-0.54	*
	Salinity Bottom	-0.62	**	-0.01		0.16		0.36	*	0.43	*	-0.03	
	DO (%) Surface	-8.70		31.65	*	-0.90		46.55	**	20.75		56.45	***
	DO (%) Bottom	0.30		-0.65		-0.55		-43.65	****	-38.45	***	0.05	
	DO (mg L^-1^) Surface	-0.53		2.19	*	-0.05		3.59	***	1.59		3.91	***
	DO (mg L^-1^) Bottom	0.03		-0.05		-0.05		-3.40	****	-2.91	***	0.01	
	Silica (mg Si L^-1^) Surface	-0.58		1.67		-0.09		-1.48		-0.57		0.10	
	Silica (mg Si L^-1^) Bottom	-0.90		0.50		-0.01		4.02		0.99		4.50	*

Two-way ANOVA and Fisher’s LSD *post-hoc* pairwise comparisons were conducted for environmental parameters in each month, where * *p* < 0.05, ** *p* < 0.01, *** *p* < 0.001 and ****p < 0.0001, blank indicates non-significance.

The first complete FGORS resurvey was carried out before aeration (July 2017) and revealed the aeration canal to have ~93% benthic FGORS coverage, with an estimated volume of 4,640 m^3^ and a mean thickness of 0.82 m (Table 3). A survey during aeration and eleven days post-hurricane Irma (September 2017) revealed an increase of 168 m^3^ of FGORS to the aeration canal following the storm (Table 3). Aeration and the aeration curtain were shut down when the hurricane passed through. In the aeration canal, the post-aeration FGORS survey indicated a mean FGORS thickness increase of 12 cm during the one-year study period, greater than our measurement precision of ± 10 cm, indicative of an actual volume change, whereas the post-aeration FGORS thickness estimate was not significantly different from pre-aeration survey in the control canal (Table 3). FGORS organic content (loss on ignition, LOI), however, was unaffected by time or treatment (Table 3, Fisher’s LSD test, *p* > 0.05 in all months).

**Table 3:** Muck coverage (area), volumes of muck and Loss on Ignition (LOI) of surface muck layers in the control and aeration canals before aeration, after Hurricane Irma and after one-year of aeration.

Canal	Sampling period	Area of FGORS (m^2^)	% of canal covered	Volume of FGORS (m^3^)	Mean FGORS thickness^a^ (m)	Change in mean FGORS thickness^b^^,^^c^	LOI of surface FGORS (%)^e^
Control Canal	Aug 2017 (Pre-aeration)	5150	87	2060	0.4	-	15.9 ± 1.26
	Sep 2017 (Post-Irma)	4288	69	1454	0.34	-15% (-6 cm)	18.2 ± 2.64
	Jun 2018 (Post-aeration)	5080	86	2250	0.44	+9% (+4 cm)	18.4 ± 1.96
Aeration Canal	Aug 2017 (Pre-aeration)	5690	93	4640	0.82	-	18.1 ± 2.49
	Sep 2017 (Post-Irma)	5836	95	4808	0.82	0% (0 cm)	19.5 ± 1.88
	Jun 2018 (Post-aeration)	6020	98	5660	0.94	+15% (+12 cm) ^d^	20.2 ± 2.81

^a^Mean FGORS thickness (m) = (volume of FGORS in m^3^)/(area of FGORS in m^2^).

^b^Change in mean FGORS thickness (%) = {[(thickness after)–(thickness before)]/thickness before} × 100%.

^c^Absolute change in mean FGORS thickness (cm) = [(thickness after in cm)–(thickness before in cm)] × 100 cm m^-1^.

^d^FGORS thickness in the aeration canal during the post-aeration survey was significantly higher compare to the pre-aeration survey (Fisher’s LSD test, *p* < 0.05).

^e^No statistical difference of surface LOI (Loss on Ignition) was found among different surveys (Fisher’s LSD test, *p* > 0.05 in all months).

## Discussion

The canal system is a nutrient- and sediment-polluted shallow brackish waterway with limited water exchange though small connections to the greater estuary. With high concentrations of dissolved nitrogen and phosphorus, high phytoplankton biomass is common. Comparing across months, temporally distinct planktonic communities developed through seasons, but communities evolved similarly in the two canals and changes seemed unrelated to treatment (Fig 4). These data showed that regional and temporal trends overshadowed would-be aeration effects. Our results were inconsistent with the findings of Huisman et al. (2004) which provided strong lab and field evidence that vertical mixing favors the growth of specific phytoplankton groups, such as diatoms [32]. One possible explanation is that the aeration system lacked the strength for sufficient mixing, especially in the summer when the water column was highly stratified [14].

During the brown tide, surface water cell counts of *A*. *lagunensis* were 35% and 50% lower in the aeration canal in April and June 2018, respectively (Fig 3A). This difference could be due to aeration canal oxidation of ammonium to chemical species not readily used by *A*. *lagunensis* [33, 34]. *A*. *lagunensis* prefer to assimilate reduced nitrogen, such as ammonium [33]. Indeed, the average concentrations of ammonium in the aeration canal were lower compared to the control canal during the bloom fading period (Fig 3H) when differences of *A*. *lagunensis* cell densities were observed (Fig 3G). Meanwhile, surface DO was significantly lower in the aeration canal, possibly being consumed in the oxidation of reduced nitrogen (Fig 3C). This suggests that aeration decreased concentrations of nitrogen available to *A*. *lagunensis*, restraining the bloom in the aerated canal. Thus, short-term aeration benefits included significantly fewer *A*. *lagunensis* during the bloom fading period (April to June 2018) (Fig 3A). Although subtle relative to seasonal shifts, the potential to impact algal bloom densities has important management implications; aeration may be useful for managing and mitigating algal blooms.

During the warm period, the deepest water (>1.5 m) in all canals was anoxic and no benthic infauna were observed (Fig 6D and Table 1). Aeration failed to maintain DO in summer bottom water when oxygen capacity is lowest and benthic respiration is highest. During the colder months (November 2017 to February 2018), however, benthic infauna were more abundant and diverse in the aeration canal (Fig 6D and 6E). FGORS thickness and surface sediment LOI was similar throughout this study, but benthic infauna communities were correlated with bottom water DO (Fig 6A). These data suggest that higher DO concentrations in the aeration canal in winter months promoted benthic infauna. Aeration apparently rendered the environment more habitable to benthic fauna without removing or changing the composition of benthic sediments, although other studies have shown that sediment organic content is a key factor for benthic infauna [35, 36]. Our finding is consistent, however, with another study showing that DO, rather than chemical pollutants, controlled benthic infauna abundance [37]. Aeration in this study facilitated colonization by benthic infauna during the colder months (< 25°C), but was insufficient to overcome bottom hypoxia during summer months (> 25°C). Benthic infauna in canals were species adapted to polluted or harsh environments. For instance, *Capitella capitata* tolerates sewage-impacted sites [38]; polychaete annelids such as *Ctenodrilus serratus* and *Capitella capitata* have high tolerance of heavy metal pollutants [39]; *Mulinia lateralis* is an opportunistic bivalve known to invade rapidly after anoxic events in polluted estuaries [40]. *Leptochelia dubia* and *Capitella capitata* were found in an aliphatic hydrocarbon polluted area [41]. Though aeration produced a habitable winter benthos, colonization was mainly by pollution-tolerant species. Higher DO promotes an overall healthier benthic system. Nevertheless, these data suggest that aeration could be employed to promote benthic colonization in areas usually characterized by hypoxic or anoxic sediments. Ecological succession might then lead to the establishment of more diverse estuarine communities over time.

Collectively, these data suggest that when aeration was able to overcome intense stratification, and it stabilized dissolved oxygen concentrations, decreased concentrations of dissolved ammonium in the water column, decreased the intensity of algal blooms and promoted benthic colonization. Based on these modest outcomes, it is possible that more efficient delivery of oxygen using other systems, such as nanobubbles, could overcome the challenges encountered during warmer months in the present study. Overall, aeration has some important management applications and may be useful for mitigating active algal blooms and promoting recovery of benthic communities. Once established, the presence of benthic communities could help to maintain dissolved oxygen in sediments via bio-irrigation and bioturbation. These processes could create a positive feedback loop, helping to sustain the improved conditions and facilitating the return of ecosystem services such as nitrogen removal via denitrification when appropriate conditions exist [42].

## Conclusion

The canal system is a highly polluted shallow brackish waterway. One year of aeration did not significantly change nutrient conditions in the form of sediment organic content, nor the inherent potential of sediments to flux nutrients into the water column and precipitate blooms. Dissolved oxygen levels and the biological communities, however, were significantly altered by aeration under some conditions. In general, aeration did not significantly change the planktonic community composition relative to the control canal. During the post-bloom period, however, aeration appeared to help weaken the brown tide, which may have been due to increased bottom DO and decreased bottom ammonium concentrations in the aeration canal. During treatment, aeration facilitated benthic animal life when temperatures dropped below 25°C, likely due to water column mixing and the increased capacity of water to hold dissolved oxygen. Aeration has important management applications and may be useful for mitigating algal blooms in flow-restricted areas and promoting benthic communities.

## Supporting information

S1 Fig Fine-grained organic-rich sediments (FGORS) survey locations (black dots) in A) the control canal, and B) the aeration canal. The maps were generated from ArcGIS Online basemap [43].
(DOCX)

S2 Fig Two-dimensional nMDS ordination plots of surface vs. bottom planktonic assemblages in aeration canal (A1, A2), control canal (C1, C2) and Grand canal (CM, AM) in different months.
(DOCX)

S1 Table Raw cell density (cells ml^-1^) of phytoplankton in each sample. (XLSX)

S2 Table Raw data of environmental parameters in each sample. (XLSX)
