# Supplementary material for: Phytoplankton and benthic infauna responses to aeration, an experimental ecological remediation, in a polluted subtropical estuary with organic-rich sediments
Source: PLoS One. 2023 Jan 24;18(1):e0280880. doi: 10.1371/journal.pone.0280880 (PMC9873162; doi:10.1371/journal.pone.0280880)
Supplement: S1 Fig — Fine-grained organic-rich sediments (FGORS) survey locations (black dots) in A) the control canal, and B) the aeration canal. The maps were generated from ArcGIS Online basemap [43]. (DOCX) [file pone.0280880.s001.docx]

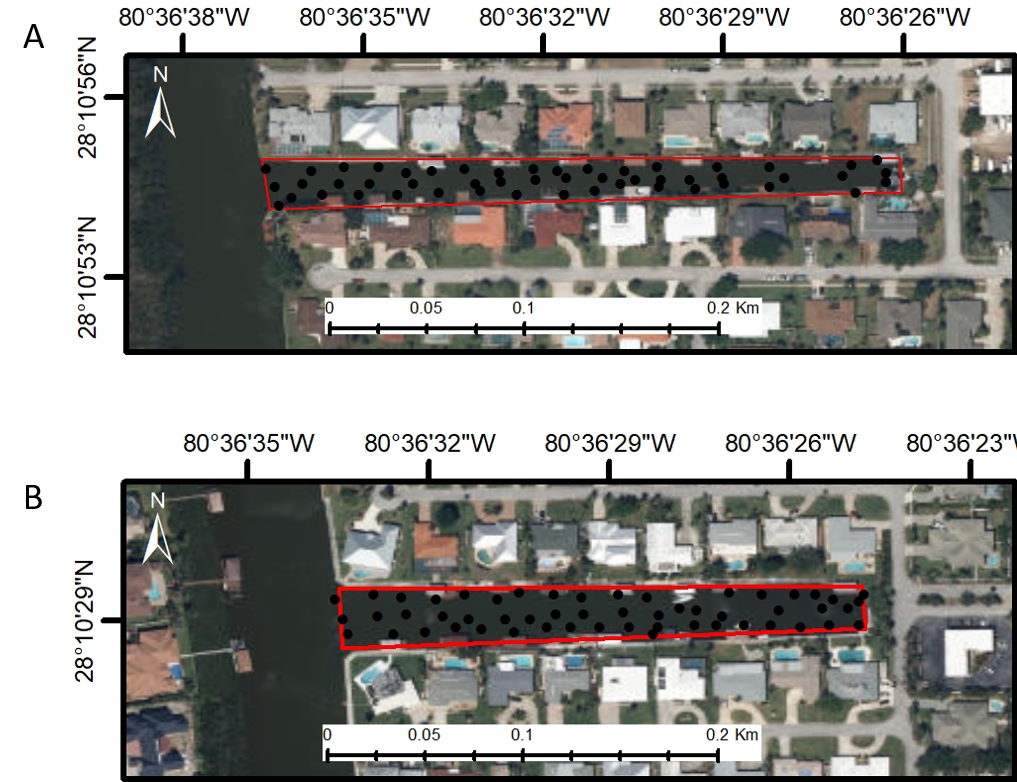


S1 Fig. Fine-grained organic-rich sediments (FGORS) survey locations (black dots) in A) the control canal, and B) the aeration canal. The maps were generated from ArcGIS Online basemap [43]
