# Supplementary material for: Phytoplankton and benthic infauna responses to aeration, an experimental ecological remediation, in a polluted subtropical estuary with organic-rich sediments
Source: PLoS One. 2023 Jan 24;18(1):e0280880. doi: 10.1371/journal.pone.0280880 (PMC9873162; doi:10.1371/journal.pone.0280880)
Supplement: S2 Fig — Two-dimensional nMDS ordination plots of surface vs. bottom planktonic assemblages in aeration canal (A1, A2), control canal (C1, C2) and Grand canal (CM, AM) in different months. (DOCX) [file pone.0280880.s002.docx]

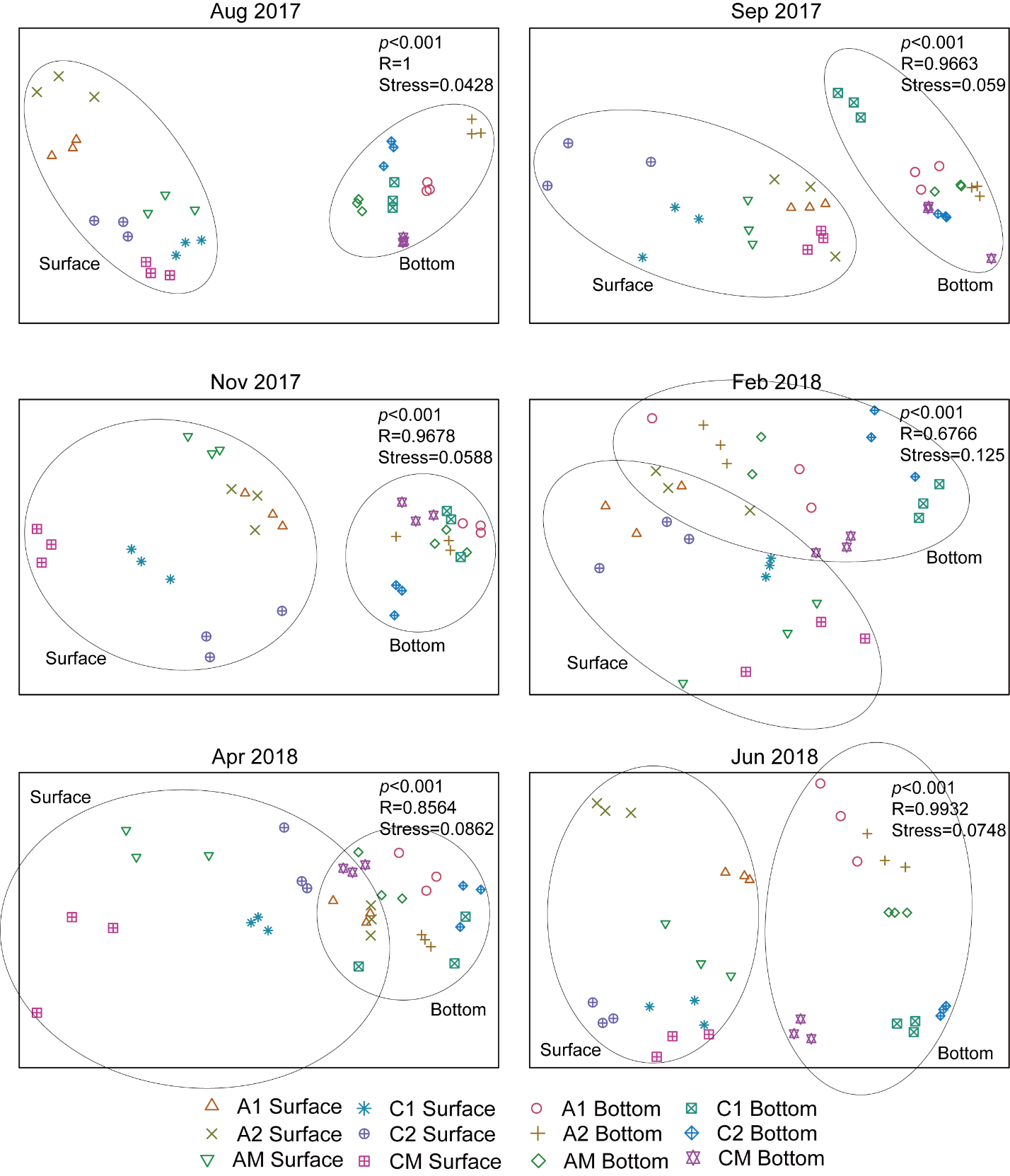


**S2 Fig.** Two-dimensional nMDS ordination plots of surface vs. bottom planktonic assemblages in aeration canal (A1, A2), control canal (C1, C2) and Grand canal (CM, AM) in different months.
